# Application of Phagotherapy in the Treatment of Burn Patients (Review)

**DOI:** 10.17691/stm2020.12.3.12

**Published:** 2020-06-28

**Authors:** А.E. Leontyev, I.V. Pavlenko, О.V. Kovalishena, N.V. Saperkin, А.А. Tulupov, V.V. Beschastnov

**Affiliations:** Researcher, Group for Thermal Trauma Study, University Clinic; Privolzhsky Research Medical University, 10/1 Minin and Pozharsky Square, Nizhny Novgorod, 603005, Russia; Junior Researcher, Group for Thermal Trauma Study, University Clinic; Privolzhsky Research Medical University, 10/1 Minin and Pozharsky Square, Nizhny Novgorod, 603005, Russia; Professor, Head of the Department of Epidemiology, Microbiology, and Evidence-Based Medicine; Privolzhsky Research Medical University, 10/1 Minin and Pozharsky Square, Nizhny Novgorod, 603005, Russia; Associate Professor, Department of Epidemiology, Microbiology, and Evidence-Based Medicine;; Junior Researcher, Group for Thermal Trauma Study, University Clinic; Privolzhsky Research Medical University, 10/1 Minin and Pozharsky Square, Nizhny Novgorod, 603005, Russia; Researcher, Group for Thermal Trauma Study, University Clinic Privolzhsky Research Medical University, 10/1 Minin and Pozharsky Square, Nizhny Novgorod, 603005, Russia

**Keywords:** burns, microflora of burn wounds, antibiotic resistance, MDR microorganisms, bacteriophages, phagotherapy in burns.

## Abstract

Treatment of patients with a burn injury is a complex process involving multicomponent multidirectional intensive therapy of the majority of organs and systems damaged by thermal effects on the skin, alternating with repeated surgical interventions aimed at removing nonviable tissues with subsequent plastic closure of wound defects. After the recovery from the burn shock, local infectious complications are considered to be the leading problem that decelerates the process of recovery and is the main cause of lethal outcomes. Since the skin integrity is broken, microorganisms penetrate readily into the internal environment of the human organism resulting in a septic state with multiple organ failure. A widespread and often uncontrollable use of antibacterial drugs in medical practice has led to the emergence of multiple drug resistance (MDR) in microorganisms.

Introduction of drugs made on the basis of bacteriophages into practice is presently becoming increasingly important. This is confirmed by the growing interest in this field of pharmacology, the development of special programs aimed at studying the processes of phage and bacterial cell interaction.

This review presents the main types of bacteria pertaining to MDR pathogens, principles of their classification, and the risk factors for infecting patients. The mechanisms of the selective action of phage particles on a bacterial cell and the possibility of using phage therapy in the treatment of burn injury (experimental and clinical data) based on the analysis of foreign literature are demonstrated as well as new positive properties of phages related to the changes in the macroorganism immune status caused by the interaction with bacteriophage particles.

## Introduction

For the last decades the lethality from burn trauma has been gradually decreasing which is mainly connected with the development of new medical equipment, achievements in the field of intensive therapy, and improvements of surgical techniques such as necrectomy and autodermotransplantation [1–3]. Nonetheless, despite the reduction of the lethality in the acute period, the improvement of survival in the remote period remains a difficult and unsolved task mainly due to sepsis and multiple organ dysfunction syndrome [4–6]. Sepsis with multiple organ failure (Sepsis-3) which is often preceded by local infectious complications is presently the main cause of death in children and adult patients with burns [7–10].

## Antibiotic resistance of microflora in burns

Since the time of their discovery at the beginning of the last century, antibiotics have been the main and often a single means of treating bacterial infections. However, eight decades of a wide and often nonselective use have led to general reduction of their efficacy. Currently, bacteria with multiple drug resistance (MDR) present a serious problem. Resistance to drugs which became known for the first time soon after the penicillin invention turned currently into a serious obstacle in the struggle against infection worldwide. According to foreign statistics, about 25,000 patients die in the European Union annually from infections caused by MRD bacteria [11, 12].

In clinical practice, wound defects are considered colonized by MDR microorganisms if cultures of *Staphylococcus aureus* resistant to methicillin, or enterococci resistant to vancomycin, and/or microorganisms resistant to extended-spectrum β-lactamases (ESBL) are isolated during inoculation [13].

Patients colonized by MDR bacteria are sources of nosocomial infection and become its potential transmitters in a hospital setting. Bearing this in mind, the American researchers [14] have studied the effects of MDR on the survival of burn patients and the length of their hospital stay as well as the role of MDR in the development of complications such as acute hepatic failure, sepsis, and multiple organ failure. The results of the study have convincingly shown that infections caused by MDR are connected with a greater number of surgical procedures, longer artificial lung ventilation, greater amount of antibiotics, and longer hospitalization. There are fully grounded concerns that serious traumas and burns may serve as an entry gate for life-threatening infectious agents as it was in the pre-antibiotic era [15].

Irrational application of antibiotics [16, 17] and their use in animal husbandry [18–20] are the most known causes of antibiotic resistance. Infectious complications are often caused by the strains of those microorganisms that a macroorganism comes across every day [21–24]. Most commonly prevention and treatment of local infection in patients with burns are especially difficult due to the impaired immune response. Besides, a complex cascade of biochemical reactions results in a “systemic apoptotic response” and, consequently, to immunosuppression which inhibits the work of normal protecting mechanisms against infection [25, 26]. A combination of the reduced immune response characterized by the decrease of the T cell function after the trauma [27, 28], the destruction of the skin barrier due to the burn injury [29, 30] enabling pathogens to spread easily over the entire organism, and long-term hospitalizations with several surgical procedures result in a higher risk of infectious complications in the burn victims. In these patients, highly virulent and stable variants of microorganisms may develop from the commensal microbiota concurrently with the impairment of their own microbiome and immune imbalance [31].

All the above said explains the following rule: if patients with a burn wound survive during the first 72 h, the most frequent cause of death at a later time is local and general infectious complications. According to the published reports [32–35], gram-positive microorganisms sensitive to antimicrobial drugs prevail in the wound defects of the burn patients in the first days of hospitalization, whereas later a more stable gram-negative microflora is detected. Such situation determines the choice of empiric antibiotic therapy in patients with severe burns.

Burn wounds are especially sensitive to infection for several reasons. Destruction of the epidermal barrier in combination with denaturation of proteins and lipids creates a favorable medium for microbial growth [36, 37]. Since it is impossible to make the required concentration of the systemically introduced antibiotics into the damaged tissues with impaired blood circulation, local application of antimicrobial preparations becomes ever more vital [38, 39].

*Pseudomonas aeruginosa* [40–42], *S. aureus*, and *Acinetobacter baumannii* [43–45] are established to be the most common etiological agents of local infectious complications in burn patients. According to Church et al. [46], *S. aureus* remains the most widespread microorganism colonizing the burn wounds in the early period. At the same time, *P. aeruginosa* is known as the most frequent cause of life-threatening infection in these patients. Both agents, especially *P. aeruginosa*, are known for their natural and acquired resistance to many antibiotics. Antibiotic-resistant strains of *P. aeruginosa* were reported to cause hospital infection outbreaks in the burn units [47–50].

A steady growth of resistance of infective agents to antibiotics associated with rendering medical aid in combination with a high susceptibility of burn wounds to infection and difficulty of systemic use of antibiotics for injured tissue debridement made the development of new effective antimicrobial medications for the treatment of burn infections one of the rapidly evolving fields of medicine. Application of bacteriophages to fight infections looks very promising [51, 52].

## Bacteriophages in treatment of burn patients

Bacteriophages, or phages, are the most common organisms on the Earth and natural controllers of bacteria abundance. They are “viruses” for bacteria and are capable of lysing, among others, the strains of virulent microorganisms irrespective of sensitivity of these strains to antibiotics. This property allows one to use bacteriophages as an alternative means of fighting infection which may be combined or interleaved with antibiotic therapy and which is able to improve the results of treating bacterial infections [53–55].

Treatment of all types of bacterial infection with bacteriophages started as early as the 20s years of the XX century. Since that time, they have been used in Russia, Georgia, Poland, and other countries [56, 57]. Russian scientists are actively studying the possibility of using bacteriophages to treat infections caused by antibiotic-resistant agents [58–62]. In recent years, a growing interest in bacteriophages has also been noted worldwide [63, 64]. One of the leading scientific organizations engaged in the study of bacteriophages remains the G. Eliava Institute of Bacteriophages, Microbiology, and Virology in Tbilisi (Georgia). A serious work on the study of phage therapy is being carried out at the Ludwik Hirszfeld Institute of Immunology and Experimental Therapy in Wroclaw (Poland). However, it should be underlined that in Europe the emergence of antibiotics with a broader spectrum of activity which are easier to produce in large quantities (i.e. commercially more profitable) has forced phage therapy to the periphery of the medical science.

Besides, in the Western medicine there are serious obstacles preventing introduction of clinical applications of phages. In the EU and the USA, discussions between the small and medium-sized pharmaceutical enterprises and the competent authorities have resulted in classifying bacteriophages as medicinal agents (biological preparations) regulated, in particular, by the European Community Code relating to medicinal products for human use (Directive 2001/83/EC of the European Parliament and of the Council of November 6, 2001). After this step, the way to licensing bacteriophages as medical products became difficult and expensive. Difficulty to obtain funding for the development of phages as medicinal agents is also a serious factor, as the problem of intellectual property protection for the types of phages (products of nature) has not been regulated. And finally, no less important is a negative perception of phage therapy which is associated with a false perception of viruses: phages are often identified (without any nuances) as “killers” and “enemies of life” [65].

According to the data presented by Cooper [66], until recently phages in the Western countries have been used to fight microbial contamination of the food products but there were no phage-based pharmacological preparations authorized to treat humans. In this connection, the main part of the clinical bacteriophage trials conducted in Europe was performed in compliance with the requirements of the Declaration of Helsinki (2013), clause 37 (Unproven Interventions in Clinical Practice).

Villareal [67] believes that phagotherapy for each individual patient should be carried out using small series of individual phage preparations. A simplified order of using bacteriophages as unregistered medicinal agents may be achieved by means of the so-called compassionate use on humanitarian grounds. This approach is acceptable for experimental therapy of severe bacterial infections in patients treated unsuccessfully with antibiotics. A notable example in this respect is Belgium where the concept of “major phages” was worked out and legislated in order to facilitate the access to phagotherapy. This approach ensures receiving of phage preparations by the patients and allows one to avoid the obstacles related to commercial production [68–70].

Despite the above-mentioned complexities, the number of cases of phage applications is increasing worldwide. In Egypt, physicians treated 30 patients with burn wounds for 5–17 days using dressings with bacteriophage solution. From 15 to 47 dressings were required for each patient [71]. This investigation was not a controlled clinical trial but it has demonstrated the safety of employing bacteriophages for the burn wound treatment. Weber-Dąbrowska et al. [72], reported about treatment of 49 burn wounds in patients infected with *P. aeruginosa*, *S. aureus*, *Escherichia coli*, *Klebsiella spp*. and/or *Proteus spp*. 42 patients recovered completely and the condition of the rest 7 patients improved notably. Jikia et al. [73] have shown successful results of treating local radiation injuries in two patients using a new biodegradable preparation capable of a slow release of phages and ciprofloxacin. The same preparation was employed in Georgia for treatment of 22 patients with chronic wounds and venous comorbid pathology after the standard therapy failure [74]. In the United Kingdom, Marza et al. reported the case of treatment of a 27-year old man with deep burns whose wounds did not close due to autodermotransplantant lysis in presence of *P. aeruginosa* infection [75]. The transplanted grafts were rapidly destroyed in spite of adequate antibiotic therapy. At the beginning of the bacteriophage treatment, there was noted a 43–1200-fold increase of microflora concentration in the wound. Three days after phagotherapy, *P. aeruginosa* from the wound was not isolated and subsequent autodermotransplantation was successful.

Presently, the therapeutic use of phages experiences the revival of renaissance in the Western world as well due to the pandemic of MDR bacteria [76]. There appeared a rapidly growing “community of phage researchers”. For instance, the first European Symposium on phagotherapy was held in Germany in 2017. In the course of the 3-day conference, the participants from 20 countries were discussing the problems relating to the phage therapy and application of phages in general. A visible result of this event was the foundation of the National Phage Forum (www.nf-phagen.de) [77].

The participants of this Symposium believe that the following factors may serve as the “pro phage” arguments [78]:

high phage specificity allows for avoiding dysbiosis in the treated media;

toxic side-effects are absent;

self-replication is restricted;

emergence of resistance to one phage does not cause generalized resistance to other phages;

phage-resistant bacteria are often less virulent;

phage reserves in nature are practically inexhaustible;

phages are effective even against MDR/ESKAPE bacteria;

phage protein-lysines can be used as an alternative;

phages are not expensive;

various ways of phage introduction are possible;

specific phage preparations can be prepared within a reasonable time.

According to the concept of personalized medicine, phage therapy is considered as the treatment responding to the concrete parameters of the human microbiome.

The following is referred to the “contra phage” arguments, i.e. probable drawbacks:

microorganisms, causative agents of the infectious process, must be uniquely identified, in the mixed infections as well;

microorganism immune system may create problems for phages;

phages reach intracellular pathogens with difficulty;

effective life/stability may vary from phage to phage which requires a regular phage titer control;

physicians must acquire new knowledge to use phage therapy in their work;

some parts of the human body may be difficult for phage application (e.g. bones, joints, deep wounds).

A number of experimental works on phagotherapy of burn wounds are presented in the scientific literature which suggests a potentially successful effect of bacteriophage application in this pathology. The experiment on mice showed that in deep burns, phage application can reduce lethality from infection caused by *P. aeruginosa* and *K. pneumoniaе* [79, 80].

The efficacy of employing bacteriophages in skin transplantation complicated by *P. aeruginosa* infection of the split-skin graft was investigated on the experimental model [81] of a burn wound in guinea pigs. It has been proved that infection of the grafts with *P. aeruginosa* destroys them while a specific lytic bacteriophage prevents the destruction during closure of the contaminated burn wound.

In the recent study by Kumari et al. [82], the efficacy of a specific bacteriophage for treatment of wound infection in mice induced by *K. pneumoniaе* has been compared with that of silver nitrate and gentamycin. In the experiment *in vivo*, a deep burn was modeled and the wound infected with *K. pneumoniae*. After the development of the infection process, the tested preparations were applied topically on the burn wound. Lethality served as a criterion for the efficacy. The results have shown that a single phage dose led to a significant lethality reduction (р<0.001). Multiple application of silver nitrate and gentamycin at a dose of 0.5% and 1000 mg/L, respectively, provided also significant protection (р<0.001). However, the level of defense provided by these two agents was lower than that of phagotherapy. The results indicate that the phage is perspective for the treatment of burn wound infections since its single topical application could reduce lethality in mice caused by *K. pneumoniae* infection compared to the multiple use of silver nitrate and gentamycin.

It was shown on the model of the burn wound in mice [83] that the lytic phage had a marked effect on *P. aeruginosa* with multiple drug resistance. Holguín et al. [84] have performed a quantitative bacteriological analysis, biofilm control, using transmission electron microscopy, gel-electrophoresis in the pulsed-field, and studied the biological activity of phage Pan70 *in vivo* on the mouse model of the burn wound. The results obtained demonstrated that the phage in relation to plankton cells and biofilms reduced considerably the bacterial population. In the main group, the survival of the animals was from 80 to 100%, being significantly different from the control (0%).

An established high specificity of phages to a bacterial cell means that in order to achieve a wide spectrum of their action, it is reasonable to use a mixture of phages [85]. In the cohort clinical study carried out by Lazareva et al. [86] it has been shown that a tableted complex pyobacteriophage provides a more rapid cure of pyoseptic complications in patients with a burn wound, temperature normalization, wound cleansing, and lethality reduction. The bacteriologic analysis of the wound discharge showed that after treatment, *Staphylococcus* and *Streptococcus* were isolated two times and *Proteus spp*. 1.5 times less frequently, while *E. coli* was not isolated at all. The number of positive hemocultures also decreased. Immune status evaluation showed statistically significant normalization of the immune response at a cellular level. The phagocytosis level remained unchanged whereas in the control group (without bacteriophage application) it became lower.

In another investigation [87], a phage cocktail was used in Klebsiella infection of the burn wound. A substantial effect of the phage cocktail was found in arresting infection process in comparison with five monovalent phages. And it was in the group of mice receiving the phage cocktail that the maximal decrease of bacterial load was registered.

Polish researchers Górski et al. [88] performed over 280 procedures with phage application in patients who were unsuccessfully treated with traditional methods. Phagotherapy appeared to be effective in 40% of patients with negative results from other types of treatment. In this group, phagotherapy resulted in complete elimination of pathogens or a steady clinical improvement. In the remaining 60% of patients the ambiguous results, absence of therapeutic effects or even worsening after the phagotherapy were noted. In the other investigations of these authors [89, 90], the obtained data have demonstrated the ability of phages (and their proteins) to suppress proinflammatory cytokines in mice and active forms of oxygen. Similar results were also noted in patients who received phage therapy [91] which suggests that phages, in addition to the commonly known antibacterial effect, may exert anti-inflammatory and immunomodulatory effects [92–94].

Scientists from Portugal [95] presented an overview on the application of bacteriophages for the control and prevention of bacterial biofilm formation. It has been noted that phages may be used separately, as a cocktail to expand the activity spectrum or in combination with other antimicrobial preparations.

Investigators from Egypt compared in the experiment *in vivo* [96] the materials for wound dressings based on the multicomponent biocompatible nanofibers with the addition of bacteriophages and without them. It has been found that the formulation with bacteriophages was observed to have a more prominent action and better wound healing while cytotoxic testing demonstrated improved biocompatibility. The authors have made a conclusion that the development of wound coverings containing bacteriophages is a promising direction.

A group of scientists from Turkey [97] has studied *in vitro* the sensitivity of resistant microorganisms, isolated from patients with complicated infections of soft tissues, to the standard bacteriophage (phage) cocktails. The results demonstrate a high potential of topical bacteriophage therapy in the management of complicated soft tissue infections.

The specialists from the Institute of Transplantation of the Warsaw Medical University (Poland) believe that the administration of phage viruses to the patients with transplantation is safe because they, unlike other viruses, do not increase the risk of transplant rejection. There is evidence that phage viruses may cause immunosuppressive effect increasing the probability of transplant engrafting. Bacteriophages inhibit considerably the T cell activation and proliferation as well as the activation of nuclear transcription factor NF-kB in response to a viral pathogen. The introduction of phage *in vivo* could decrease cellular infiltration of the skin allograft. The data presented in work [98] have shown that phages can be used in clinical transplantology to treat infections caused by resistant bacteria and be conceivably a supplementary means of immunosuppressive therapy. Some researchers believe that bacteriophage production will soon become one of the leading branches of the pharmaceutical industry [99].

An interesting project for investigating the potentials of using bacteriophages for burn wounds is a randomized controlled study PhagoBurn (www.phagoburn.eu) which was funded by the European Commission and was launched in 2013 with participation of France, Belgium, and Switzerland. The project allowed the scientists to evaluate the phage therapy efficacy in the treatment of burn wounds infected with *P. аeruginosa* [100].

## Conclusion

Bacteriophages are confidently taking one of the leading places in the row of antibacterial preparations and phagotherapy becomes a component of successful management of patients with MDR infections. The increasing number of publications undoubtedly reflects a growing interest of specialists from different countries to the application of bacteriophages in the treatment of complicated wounds including those in burn patients. The available data illustrate both significant advances of the Russian scientists in the exploration and application of bacteriophages in clinics and accelerated development of investigations of our western colleagues. Presently, the evidence base in relation to a therapeutic potential of bacteriophages in treating burn patients has been formed primarily by the described cases of their successful applications in clinical practice, experimental studies *in vitro* and *in vivo*, and is oriented in many respects to the expert opinion. It seems extremely important to continue investigations of the possibility of application and efficacy of phagotherapy in the treatment of burn wound infections within the frames of epidemiological and clinical studies with a more sophisticated design.
